# A two-echelon location routing problem considering sustainability and hybrid open and closed routes under uncertainty

**DOI:** 10.1016/j.heliyon.2023.e14258

**Published:** 2023-03-07

**Authors:** Masoud Hajghani, Mohammad Ali Forghani, Ali Heidari, Mohammad Khalilzadeh, Omid Kebriyaii

**Affiliations:** a Department of Industrial Engineering, Shahid Bahonar University of Kerman, Iran; b Faculty of Management and Economics, Shahid Bahonar University of Kerman, Iran; c Department of Industrial Engineering, Iran University of Science and Technology, Tehran, Iran; d CENTRUM Católica Graduate Business School, Lima, Peru. Pontificia Universidad Católica del Perú, Lima, Peru

**Keywords:** Location-routing, Greenhouse gas emission, Multi-objective optimization, Augmented Epsilon Constraint (AEC), NSGA-II, MOSFA

## Abstract

Location-routing is an extremely important problem in supply chain management. In the location-routing problem, decisions are made about the location of facilities such as distribution centers as well as the set of vehicle routes. Today, organizations seek to reduce the transportation cost by outsourcing leading to a particular kind of transportation problems known as open routing. However, the increasing attention to environment have led to paying more attention to environmental issues and reducing the environmental impacts of logistics activities. To this end, in this paper, both open and closed routes were simultaneously addressed by developing a multi-objective mixed integer linear programming model that included three economic, environmental, and social responsibility aspects. The three objective functions of the proposed model encompass the minimization of total costs and greenhouse gas emissions, and the maximization of employment rate and economic development. Also, in this study, a different type of routing was considered in each echelon. A small-sized problem instance was solved using the Augmented Epsilon Constraint (AEC) method with the CPLEX Optimizer Solver for the validation of the proposed model. Moreover, the sensitivity analysis was performed to investigate the effect of changing main parameters on the values of the objective function. Due to the NP-Hardness of the problem, two efficient metaheuristic algorithms of Non-dominated Sorting Genetic Algorithm (NSGA-II) and Multi-Objective Stochastic Fractal Search (MOSFS) were exploited to solve the medium and large size problems. The performance of the algorithms was compared on the basis of six different well-known indexes of Time, MID, RAS, Diversity, Spacing, and SNS. According to the obtained results, the performance of the MOSFS algorithm was %20, %9, %11.22, %10.03, and %19.06 higher than the performance of the NSGA-II on the basis of SNS, RAS, MID, Diversity, and Time indexes, respectively. On the other hand, the NSGA-II performance was %6.3 higher than the MOSFS performance in terms of Spacing index.

## 1 Introduction

To take advantage of competitive and economic advantages, it is essential for the organization to focus on minimizing costs or maximizing profits [1]. On the other hand, the production activities have detrimental environmental impacts due to the overconsumption of natural resources [2]. Road transportation also faces remarkable environmental problems and issues such as the reduction of fossil fuels usage and greenhouse gases emission. To resolve these issues, governments and organizations must take practical initiatives and change transportation policies to provide sustainable logistics operations [3]. The environmental impacts have been considered as one of the primary aspects of sustainable development due to raising awareness about the pollutants and their negative impacts on humans’ lives [4].

Sustainable development also addresses social responsibility issues [5]. Sustainable development is an initial strategic goal for the supply chain [6]. Most of the previous studies on sustainability problems have investigated the economic and environmental aspects. However, limited studies have addressed the social responsibility aspect. This lack of research is due to the complexity of social responsibility modeling [7].

Sustainable supply chain problems are classified into two main categories: green and sustainable supply chain [8] so that the green supply chain (the first category) is the subset of sustainable supply chain (the second category) [9]. A supply chain consists of suppliers, manufacturing plants, and customers as well as materials flow, information flow and cash flow between the components. The management of a supply chain system can be divided into three levels: strategic, technical, and operational levels. An effective cooperation among the entire supply chain system can lead to a significant reduction in total costs. Supply chain network design usually initiates with selecting potential locations and determining the capacity required for factories [10]. Leng et al. proposed a bi-objective model for the sustainable location-routing problem and solved it using well-known multi-objective evolutionary algorithms [11]. In addition, Galindres et al. [12] considered a multi-objective sustainable capacitated location routing problem and solved it using exact and approximate solution methods.

A considerable amount of investment is required for setting up new facilities (factories, depots, or warehouses). However, these facilities can be utilized for a lengthy duration [13]. The performance of the entire supply chain network is considerably influenced by the locations of factories. Location-routing problems (LRP) integrate two basic problems in logistics. In LRP, the decisions on the locations of various facilities are integrated with the decisions on the vehicles’ routes. Independent decisions on either of these two categories can significantly affect the global optimal solution [14].

Delivery of goods from origin to destination is often done through one or more intermediate facilities such as hubs and warehouses. These types of distribution systems are commonly known as multi-echelon systems. Each echelon can be defined as the connector between two adjacent levels. Here, an echelon may be any kind of facility. Multi-echelon distribution systems are often used by public and private sector organizations in distribution networks to implement transportation systems and traffic planning strategies. The common examples of multi-echelon distribution systems are logistics systems and multi-mode urban transportation systems. Two-echelon distribution systems are a special type of multi-echelon distribution systems in which the distribution network consists of three levels. Two-echelon distribution systems have received more attention in recent years because of their wide application in daily jobs.

**Fig. 1:**
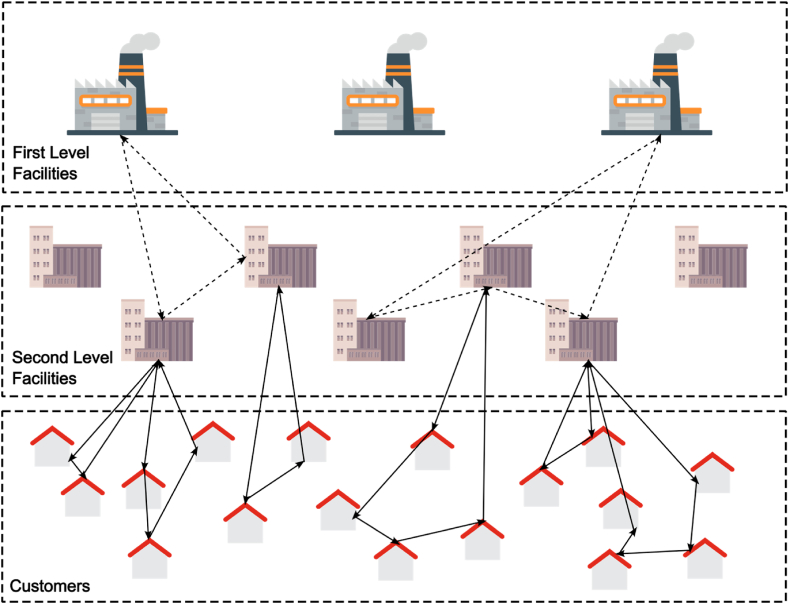
An overview of a two-echelon location-routing problem (2E-LRP).

A distribution network that consists of three distinct sets of vertices corresponding to potential factory locations (origins), potential secondary (mid-level) facility locations and destinations (customers) is known as two-echelon distribution network, in which the locations of customers are constant and predetermined, however, the locations of the required plants and/or the peripheral and secondary provisions and facilities are not predefined. Fig. 1 depicts an overview of a two-echelon location-routing problem (2E-LRP).

Nowadays, transportation companies often outsource transportation in one or more echelons to decrease the transportation cost and consequently the cost of the final product. This matter has led to the emerge of a new type of vehicle routing problems called open routing (The vehicles do not return to the origin after serving the last customer and an open route is created). In practice, the open vehicle routing problem (OVRP) relates to a situation in which a company does not possess its own fleet of vehicles or its current fleet of vehicles is not able to meet the demands of all customers. In this case, all or a part of the distribution activities are contractually outsourced to a third-party logistics company [15].

Nevertheless, the reduction of the adverse and negative effects of transporting operations and activities on the environment is a critical issue. Consequently, green transportation systems have been developed in manufacturing and distribution industry. Different approaches such as Pollution Routing Problem (PRP) may be employed in the vehicle routing problems for reducing the greenhouse gases emissions. The goal of PRP is to select the vehicle routes with the lowest greenhouse gas emissions, especially carbon dioxide emissions [7].

According to the aforementioned issues, this research seeks to present a sustainable three-objective model for 2E-LRP under uncertainty. The problem consists of different three levels including factories, depots, and customers. In addition to the minimization of the total costs of the whole distribution system, the amount of CO_2_ emissions is minimized, which is one of the major criteria and factors for sustainable development. Also, the social responsibility aspect (employment rate and the community development) is considered in the proposed model. In this problem, the location of depots is determined and the routing of the vehicles is distinguished on both two echelons. In addition, both open and closed vehicle routing problems are taken into account. The proposed model is called the hybrid open and closed sustainable two-echelon location-routing problem (COM-S2ELRP). The results of solving this multi-objective mathematical programming model determine the optimal location of the depots, the routing of the vehicles, and the optimal amount of transportation in both two echelons, in addition to the allocation of customers and depots.

## 2 Literature review

Routes are expanded from the initial facilities to the depots and from the depots to the customers to hand over goods to the customers. In this type of LRP, routes from the initial facilities to the depots are called the first echelon routes, and routes from the depots to the customers are called second the echelon routes [16]. Jacobsen & Madsen investigated 2E-LRP for the first time considering the newspaper distribution system in Denmark [17]. Lin and Lei [18] developed a 2E-LRP model that included a number of depots as well as two kinds of customers. Crainic et al. [19] solved 2E-LRP using a heuristic algorithm. Nguyen et al. [20] suggested a model for 2E-LRP presuming a focal depot and various potential locations for other depots. Also, the set-up costs of depots were different and their capacities were considered limited.

Martínez-Salazar et al. [21] presented a bi-objective 2E-LRP model including cost and traveled distance minimization and solved it using two metaheuristic algorithms. Also, Rahmani et al. [22] developed a mixed integer programming (MIP) model with several assumptions for 2E-LRP and solved it employing two heuristic approaches. Moreover, Vidović et al. [23] provided a MIP model for 2E-LRP to design a collecting and recycling system for the non-hazardous recyclable waste. Ouhader and El kyal [24] suggested a sustainable 2E-LRP for shipping products from suppliers to customers. The model included three objective functions: minimization of emissions and costs as well as maximization of job opportunities. Finally, the Epsilon Constraint method (ECM) was exploited as a solution method. Zhao et al. [23] considered a heterogeneous transportation fleet for 2E-LRP and exploited a heuristic approach to tackle it.

Pichka et al. [25] introduced a new type of 2E-LRP known as the two-echelon open location-routing problem (2E-OLRP) presuming the vehicles do not turn back to the depots. Darvish et al. [26] proposed a flexible 2E-LRP considering flexible delivery time and distribution network design. Amiri et al. [27] considered time window for 2E-LRP and utilized the Lagrangian Relaxation method as the solution approach.

Cao et al. [28] reviewed the studies on 2E-LRP which shows there is no research on open and closed routing problems. Lu et al. [29] proposed a 2E-LRP model considering closed routing and applied two metaheuristic algorithms for solving the model. Liu and Jiang [30] developed an MIP model for the combination of open and closed vehicle routing problem (COMVRP) and employed a metaheuristic algorithm to deal with it. Yu and Lin [31] applied the Simulated Annealing (SA) algorithm to solve the open vehicle routing problem.

Several studies conducted on the sustainable vehicle routing problems have addressed economic, environmental, and social responsibility aspects. For example, Navazi et al. [32] incorporated sustainability into location-routing problem considering economic (cost minimization), environmental (emission minimization) and social responsibility (customer satisfaction) aspects for collecting expired products. They solved the problem using NSGA-II and MOPSO. Also, Navazi et al. [33] proposed a sustainable location-routing model for product delivery to customers. In this study, four goals of reducing costs, environmental pollutions, driving accidents and increasing customer satisfaction through timely delivery were considered. Tayebi Araghi et al. [34] developed a model for the green multi-facilities open location-routing problem with planar facility locations and uncertain customer. In this model, the random location of facilities was included as an effective factor in supply chain costs. Also, the location of the depots, the allocation of vehicles and the selection of the routes were taken into account to minimize the emission of carbon dioxide in the entire supply chain. Nucamendi-Guillén et al. [35] suggested a mixed integer linear programming (MILP) model for the multi-depot open location-routing problem with a heterogeneous fixed fleet (MD-OLRP) to solve the problem of a company in collecting raw consumable materials. In addition, Momeni et al. [36] used vehicle routing in environmental protection problem for the first time.

Liu and Liu [37] suggested a sustainable two-stage stochastic model. Zhang et al. [38] provided a sustainable location-routing model considering multiple depots in emergency conditions to minimize relief cost, traveling time, and CO_2_ emission. Nekooghadirli et al. [39] presented a bi-objective model for the location-routing and inventory problem considering uncertain traveling time and customer demand. They also utilized several metaheuristic algorithms to deal with the problem. Tang et al. [40] introduced a model for the sustainable location-routing and inventory problem. In this study, environmental issues based on consumer behavior were investigated. Ebrahimi [41] proposed a model with multiple objective functions for random allocation routing problem taking the concepts of discount and sustainability into account. The objectives comprise economic (minimizing costs), environmental (minimizing pollutions), and social responsibility (maximizing responsiveness to customers) aspects. Finally, the model was solved using ECM.

Wang et al. [42] modelled 2E-LRP considering multiple periods and shared transportation resource to find optimal facility locations as well as optimal routes in different periods during the decision horizon. Gandra et al. [43] investigated the impact of loading restrictions on the two-echelon location-routing problem. Fallahtafti et al. [44] presented a multi-objective two-echelon location-routing problem for cash logistics to reduce the risk of theft while transporting cash and solved the problem using meta-heuristic approaches. Bassey and Zelibe [45] suggested a mixed integer nonlinear programming model for the problem of two-echelon inventory location model with response time requirement and lateral transshipment. Cheng et al. [46] proposed a multi-period two-echelon location-routing model (MP-2ELRP) to minimize the cost and duration of cleaning up the waste caused by accidents using Temporary Disaster Waste Management Sites (TDWMSs). In these sites, waste was stored and processed before being sent to disposal sites. Liu et al. [47] examined the sustainable location, routing, and inventory problem. Moreover, Babaee et al. [48] addressed the sustainable location routing problem. Furthermore, Masoudipour et al. [49] presented a model for the sustainable closed-loop supply chain problem. Du et al. [50] presented a multi-objective optimization model for two-echelon joint delivery location-routing problem considering carbon emissions and operational costs under online shopping. Mahmoodirad et al. [51] investigated the sustainable multi-objective multi-product location-routing-inventory problem considering open routing and direct transportation. They also employed several metaheuristic algorithms such as NSGA-II and MOGWO. Zandkarimkhani et al. [52] developed a model for designing a sustainable open loop supply chain network considering the location-routing problem with a combined approach based on Fuzzy AHP and Fuzzy TOPSIS methods. Raeisi and Jafarzadeh Ghoushchi [53] developed a robust fuzzy multi-objective location-routing model for hazardous waste problem under uncertain conditions to overcome waste disposal and prevent the spread of COVID-19. They solved the model using meta-heuristic algorithms and compared the solutions. In addition, Ben Mohamed et al. [54] suggested a model for the two-echelon stochastic multi-period capacitated location-routing problem (2E-SM-CLRP) considering stochastic and time-varying demand and varying costs.

The review of studies on the two-echelon location-routing problems (2E-LRP) shows that open and closed routes have not been considered simultaneously in previous studies. In this paper, a sustainable mixed integer linear programming (MILP) model including economic aspect (cost minimization), environmental aspect (minimization of transportation emissions) and social responsibility aspect (job creation and community development) is proposed for the problem of two-echelon location-routing considering the hybrid open and closed routes under uncertainty conditions. In addition, in this research, a distinct kind of routing is examined in each echelon.

## 3 The proposed mathematical model

As stated in the previous section, both open and closed routes have not been simultaneously investigated so far. In the present research, a sustainable MILP model consisting all three aspects of economic (cost minimization), environmental (minimization of transportation emissions) and social responsibility (job creation and community development) is rendered for 2E-LRP taking the hybrid open and closed routes under uncertainty conditions into account. Also, a different type of routing is considered in each echelon.

This section proposes a three-objective MILP model for COM-S2ELRP under uncertainty conditions. In the proposed model, the minimization of total costs and greenhouse gas emissions together with the maximization of employment rate and community development through establishing the facilities in deprived and undeveloped regions is considered.

The real-world assumptions are incorporated into this model in such a way that the closed routes are assumed for the first echelon (each trip should finish at the beginning point that is a factory) and the open routes are assumed for the second echelon (succeeding the satisfaction of the last customer's demand, the vehicle do not turn back to the depot, in other words, the fleet is outsourced based on a contract for transporting goods).

**Fig. 2:**
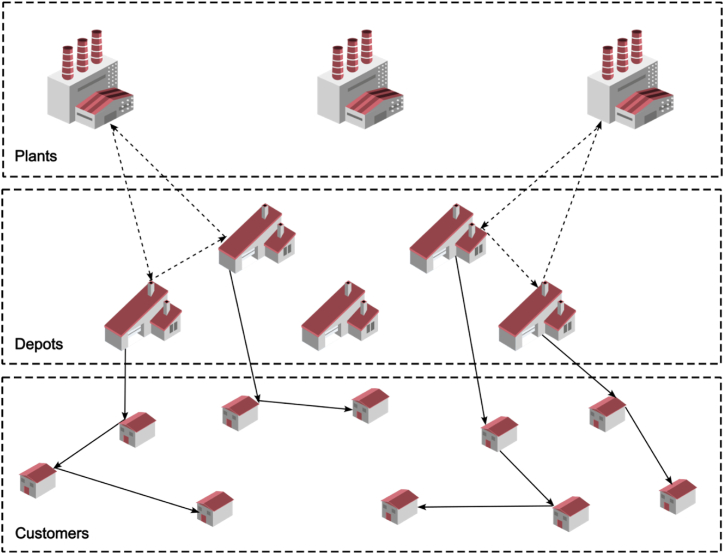
An example of a feasible solution to an instance problem.

Fig. 2 shows an overview of a feasible solution to an instance single-period single-product problem.

As shown in the above figure, decision on the facility location is made only at the second level, and at the first level decision on the location is made, while decision on routing is made in both echelons.

The sets, indices, parameters, and decision variables of the proposed three-objective MILP model are presented as follows:

### 3.1 Sets and indices

**Table:** 

N	$N=N_{p}\cup N_{d}\cup N_{c}$: The set of the whole network notes
$N_{p}$	The set of factories at the first level of the distribution network
$N_{d}$	The second level facility set (depots, warehouses or distribution centers)
$N_{c}$	The set of customers at the third level
A	The set of all connecting arcs between network nodes
V	The set of available vehicles in each facility (factory) of the first level of the distribution network
i,j,k,m	The index of each network node
v	The vehicle index in the first echelon

### 3.2 Parameters

#### 3.2.1 Parameters related to the economic aspect

**Table:** 

${FD}_{k}$	The fixed cost of setting up intermediate facilities (depots)
${FV}_{1}^{v}$	The fixed cost of using each vehicle in the first echelon
${FV}_{2}$	The fixed cost of using each vehicle in the second echelon
$d_{i}$	The demand of customer *i*
$c_{ij}$	Direct (Euclidean) distance (cost corresponding to transport) between two nodes *i* and *j*∈*N*
NV	Maximum number of available vehicles in each depot
${CP}_{k}$	The capacity of the first level facility (factory)
${CD}_{k}$	The capacity of the second level facility (depot)
${CV}_{1}^{v}$	The capacity limit of each vehicle in the first echelon
${CV}_{2}$	The capacity limit of each vehicle in the second echelon

#### 3.2.2 Parameters related to the environmental aspect

**Table:** 

$E_{ve1}^{v}$	The amount of CO_2_ emissions of the empty vehicles of the first echelon (kg CO_2_/km)
$E_{ve2}$	The amount of CO_2_ emissions of the empty vehicles of the second echelon (kg CO_2_/km)
$E_{vf1}^{v}$	The amount of CO_2_ emissions of the full vehicles of the first echelon (kg CO_2_/km)
$E_{vf2}$	The amount of CO_2_ emissions of the full vehicles of the second echelon (kg CO_2_/km)

#### 3.2.3 Parameters related to the social responsibility aspect

**Table:** 

$\omega_{em}$	The importance factor of employment
$\omega_{ed}$	The importance factor of community development
${JO}_{k}$	Number of permanent jobs created corresponding to each depot in node (region) *k*
${EV}_{k}$	The rate of increase in the economic value of node *k* corresponding to a depot set-up in node (region) *k*
${ur}_{k}$	The unemployment rate of node (region) *k*
${rd}_{k}$	The level of economic development in node (region) *k*

### 3.3 Decision variables

**Table:** 

	Binary variable corresponding to depot set-up (1: if depot *k* is set up, otherwise: 0)
$s_{ij}^{v}$	Binary variable corresponding to routing in the first echelon (1: if vehicle v is traveled between nodes *i* and *j* in the first echelon, otherwise: 0)
$x_{ij}$	Binary variable corresponding to routing in the second echelon (1: if a route is created between nodes *i* and *j* in the second echelon, otherwise: 0)
$w_{ik}$	Binary variable corresponding to the assignment of customers to depots (1: if customer *i* is assigned to depot *k*, otherwise: 0)
$Q_{ij}^{v}$	The integer variable corresponding to the amount of remaining load in vehicle *v* in the first echelon traveling from node *i* to node *j*
$U_{ij}$	The integer variable corresponding to the amount of remaining load in the vehicles of the second echelon traveling from node *i* to node *j*

Consider a complete directional graph network G=(N,A). In which, $N=N_{p}\cup N_{d}\cup N_{c}$ represents the set of all network nodes. $N_{p}$, $N_{d}$, and $N_{c}$ denote factories (the first level), potential locations for depots (the second level), and customers (the third level), respectively. Also, A={(i,j):i,jεN,i≠j} denotes the set of all network arcs. In addition, the following relation exists for all *i*, *j*, and *d* ∈ N:

$c_{id}+c_{dj}\geq c_{ij}$, in which, $c_{ij}$ represents the cost of traveling between nodes *i* and *j*. Every customer has a demand equal to the amount of $d_{i}$: $0\leq d_{i}\leq {CV}_{2}$.

### 3.4 Three-objective mixed integer linear programming (MILP) model

Now, the three-objective MILP model is presented for the COM-S2ELRP problem in hand:

(1) $$Minimize\;Z_{1}=\sum_{i\in N_{p}\cup N_{d}}\sum_{j\in N_{p}\cup N_{d},\left(i\neq j\right)}\sum_{v\in V}c_{ij}s_{ij}^{v}+\sum_{i\varepsilon N/N_{p}}\sum_{j\in N_{c}}c_{ij}x_{ij}+\sum_{k\in N_{d}}{FD}_{k}y_{k}+\sum_{i\in N_{p}}\sum_{j\in N_{d}}\sum_{v\in V}{FV}_{1}^{v}s_{ij}^{v}+\sum_{i\varepsilon N_{c}}\sum_{k\in N_{d}}{FV}_{2}x_{ki}$$

Equation (1) minimizes the total cost of the entire system and comprises five components: (1) The transportation costs of the first echelon vehicles, (2) The transportation costs of the second echelon vehicles, transportation costs of second-class travel by secondary vehicles, the fixed costs of setting up depots in the second level, the fixed costs of employing vehicles in the first echelon, and the fixed costs of utilizing vehicles in the second echelon.

(2) $$\mathrm{Min}\;imize\;Z_{2}=\sum_{i\in N_{p}\cup N_{d}}\sum_{j\in N_{p}\cup N_{d}}\sum_{v\in V}c_{ij}\left[\left(\left(E_{vf1}^{v}-E_{ve1}^{v}\right)\frac{Q_{ij}^{v}}{{CV}_{1}^{v}}\right)+E_{ve1}^{v}s_{ij}^{v}\right]+\sum_{i\varepsilon N/N_{p}}\sum_{j\in N_{c}}c_{ij}\left[\left(\left(E_{vf2}-E_{ve2}\right)\frac{U_{ij}}{{CV}_{2}}\right)+E_{ve2}x_{ij}\right]$$

Equation (2) minimizes the total amount of CO_2_ emissions of the whole system and consists of two components: the amount of CO_2_ emissions of the first echelon, and the amount of CO_2_ emissions of the second echelon. It is noteworthy that the amount of CO_2_ emissions in both two echelons depends on the type of vehicle and its load and distance traveled, also, the vehicles are heterogeneous in the first echelon and homogeneous in the second echelon.

The third objective function deals with social responsibility. According to ISO 26000 guidance on social responsibility, community participation and development is one of the main aspects of social responsibility. There are two main methods to calculate the social responsibility aspect:

(1) Employment,
(2) Regional development

In the proposed model, setting up a depot as a facilitator of intermediate transfer can lead to the participation and development of the community. In fact, employment and economic development are the two main reasons for establishing facilities. In other words, job opportunities resulting from a depot set-up are considered. Social value is calculated using the number of created job opportunities and the unemployment rate in that region. In this regard, creating job opportunities in a region with a higher unemployment rate leads to greater social value. Regional development rate and economic value of established depots are addressed to assess social value. Hence, higher social value means more importance given to the less developed regions. The economic development and employment criteria are presented as follows:

(3-1) $${SI}_{em}=\sum_{k\in N_{d}}{\left({JO}_{k}\right)ur}_{k}y_{k}$$

(3-2) $${SI}_{ed}=\sum_{k\in N_{d}}{EV}_{k}\left(1-{rd}_{k}\right)y_{k}$$

Both above criteria are taken into account in the third objective function of the model shown in Equation (3):

(3) $$\mathrm{Max}\;Z_{3}=\omega_{em}\left(\sum_{k\in N_{d}}{\left({JO}_{k}\right)ur}_{k}y_{k}\right)+\omega_{ed}\left(\sum_{k\in N_{d}}{EV}_{k}\left(1-{rd}_{k}\right)y_{k}\right)$$

Subject to:

Constraints related to the first echelon:

(4) $$\sum_{k\in N_{p}}y_{k}=\left|N_{p}\right|$$

Equation (4) ensures that the $N_{p}$ number of factories must be established at the first level.

(5) $$\sum_{j\in N_{d}}s_{ij}^{v}\leq 1\forall i\in N_{p},\forall v\in V$$

Constraint (5) states that each of the available vehicles in each factory cannot travel more than one route.

(6) $$\sum_{\begin{array}{c} j\in N_{p}\cup N_{d}\\ \left(j\neq i\right) \end{array}}s_{ij}^{v}=\sum_{\begin{array}{c} j\in N_{p}\cup N_{d}\\ \left(j\neq i\right) \end{array}}s_{ji}^{v}\forall i\in N_{p}\cup N_{d},\forall v\in V$$

(7) $$\sum_{\begin{array}{c} j\in N_{p}\cup N_{d}\\ \left(j\neq k\right) \end{array}}\sum_{v\in V}s_{kj}^{v}=y_{k}\forall k\in N_{d}$$

(8) $$\sum_{\begin{array}{c} j\in N_{p}\cup N_{d}\\ \left(j\neq k\right) \end{array}}\sum_{v\in V}s_{jk}^{v}=y_{k}\forall k\in N_{d}$$

Constraints (6), (7), and (8) ensure that for each node in the first and second levels corresponding to the first echelon routes, exactly one arc with one vehicle enters and one arc with the same vehicle exits.

(9) $$\sum_{v\in V}s_{ik}^{v}\leq y_{k}\forall i\in N_{p},\forall k\in N_{d}$$

Constraint (9) ensures that the first echelon routes originating from the existing factories go to a depot that already has been set up.

(10) $$\sum_{j\in N_{d}}\sum_{v\in V}Q_{ij}^{v}\leq {CP}_{i}\forall i\in N_{p}$$

Constraint (10) shows that the total demand met by the factories in the first echelon cannot exceed their capacity.

(11) $$s_{ij}^{v}=0\forall i,j\in N_{p},\forall v\in V$$

Constraint (11) hinders the formation of a route between factories in the first echelon.

(12) $$\sum_{j\in N_{p}\cup N_{d}}\sum_{v\in V}Q_{ji}^{v}-\sum_{j\in N_{p}\cup N_{d}}\sum_{v\in V}Q_{ij}^{v}=\sum_{k\in N_{c}}d_{k}w_{ki}\forall i\in N_{d}$$

Constraint (12) refers to two basic principles in the first echelon: first, that it guarantees the balance of flow in the routes created in the first echelon, and second, that this constraint hinders the formation of sub-loops in the first echelon.

(13) $$Q_{ij}^{v}\leq {CV}_{1}^{v}s_{ij}^{v}\forall i,j\in N_{p}\cup N_{d}\;\left(i\neq j\right),\forall v\in V$$

Constraint (13) ensures that the total load of a vehicle in the first echelon cannot exceed the capacity of that vehicle.

(14) $$\sum_{i\in N_{d}}\sum_{j\in N_{p}}Q_{ij}^{v}=0\forall v\in V$$

Constraint (14) mandates that the first echelon vehicle returning to the origin factory at the end of the tour (closed route) cannot have any load and must be empty.

Constraints related to the second echelon:

(15) $$x_{kj}\leq y_{k}\forall j\in N_{c},\forall k\in N_{d}$$

Constraint (15) indicates that the second echelon routes can originate from a depot that already has been set up.

(16) $$\sum_{j\varepsilon N/N_{p}}x_{ij}=1\forall i\in N_{c}$$

Constraint (16) ensures that each customer receives service exactly once.

(17) $$\sum_{j\varepsilon N/N_{p}}x_{ij}=\sum_{j\varepsilon N/N_{p}}x_{ji}\forall i\varepsilon N/N_{p}$$

Constraint (17) ensures that for each node in the second echelon the number of incoming arcs (routes) is equal to the number of out coming arcs (routes).

(18) $$\sum_{j\varepsilon N/N_{p}}U_{ji}-\sum_{j\varepsilon N/N_{p}}U_{ij}=d_{i}\forall i\in N_{c}$$

Constraint (18) denotes the flow balance in the second echelon.

(19) $$U_{ij}\leq {CV}_{2}x_{ij}\forall i,j\in N/N_{p}\;\left(i\neq j\right)$$

Constraint (19) guarantees that the remaining demand of the customers (the remaining load of the vehicle in the second echelon) cannot exceed the capacity of the vehicle.

(20) $$\sum_{j\in N_{c}}U_{kj}=\sum_{j\in N_{c}}w_{jk}d_{j}\forall k\in N_{d}$$

Constraint (20) mandates that the total demand of customers assigned to a particular depot must be met by vehicles sent from the same depot.

(21) $$\sum_{j\in N_{c}}U_{jk}=0\forall k\in N_{d}$$

Constraint (21) ensures that in the second echelon the amount of remaining load in the vehicle after serving the last customer is equal to zero (open route).

(22) $$U_{ij}\leq \left({CV}_{2}-d_{i}\right)x_{ij}\forall i\in N_{c},\forall j\in N/N_{p}$$

(23) $$U_{ij}\geq d_{j}x_{ij}\forall i\in N/N_{p},\forall j\in N_{c}$$

Constraints (22) and (23) define the upper and lower bounds for the flow variables in the second echelon, respectively.

(24) $$\sum_{k\in N_{d}}w_{ik}=1\forall i\in N_{c}$$

Constraint (24) mandates that each customer is assigned to only one depot.

(25) $$\sum_{i\in N_{c}}{d_{i}w}_{ik}\leq {CD}_{k}y_{k}\forall k\in N_{d}$$

Constraint (25) ensures that the total demand of customers assigned to a depot cannot exceed the capacity of that depot.

(26) $$x_{ik}\leq w_{ik}\forall i\in N_{c},\forall k\in N_{d}$$

(27) $$x_{ki}\leq w_{ik}\forall i\in N_{c},\forall k\in N_{d}$$

(28) $$x_{ij}+w_{ik}+\sum_{\begin{array}{c} m\in N_{d}\\ \left(m\neq k\right) \end{array}}w_{jm}\leq 2\forall i,j\in N_{c}\;\left(i\neq j\right),\forall k\in N_{d}$$

(29) $$\sum_{i\in N_{c}}x_{ki}\leq NV\forall k\in N_{d}$$

Constraints (26–29) prevent the creation of impractical routes as well as sub-loops in the second echelon.

(30) $$s_{ij}^{v}\in \left\{0,1\right\}\forall i,j\in {N/N}_{c},\forall v\in V$$

(31) $$x_{ij}\in \left\{0,1\right\}\forall i\in {N/N}_{p},\forall j\in N_{c}$$

(32) $$y_{k}\in \left\{0,1\right\}\forall k\in N_{d}$$

(33) $$w_{ik}\in \left\{0,1\right\}\forall i\in N_{c},\forall k\in N_{d}$$

(34) $$Q_{ij}^{v}\geq 0\forall i,j\in {N/N}_{c},\forall v\in V$$

(35) $$U_{ij}\geq 0\forall i\in {N/N}_{p},\forall j\in N_{c}$$

Constraints (30–35) define the decision variables of the model.

### 3.5 The proposed robust possibilistic programming model

Because of the insufficiency of information and uncertainty associated with the model parameters in the previous section, determining the exact values of the parameters will be very difficult and impossible in some case. The model presented in the previous section for the sustainable location-routing problem is a multi-objective mixed integer programming model in which the fuzzy numbers are utilized for the non-deterministic cost and capacity parameters. To deal with the uncertainty of the problem, a robust possibilistic programing model is developed. Possibilistic programming is utilized to tackle the uncertainty associated with the objective function coefficients and the model constraints. In possibilistic programming, the historical data are considered together with the decision-maker's opinion. In this study, a robust possibilistic programming model based on the chance-based possibilistic programming model presented by Pishvaee et al. [55].

A solution to an optimization problem is a robust solution if it encompasses feasibility robustness and optimality robustness. Feasibility robustness means that the solution must remain feasible for almost all possible scenarios of uncertain parameters. Optimality robustness means that the value of the objective function for a robust solution must have a minimum deviation from its optimal value for almost all amounts of uncertain parameters.

### 3.6 The proposed robust programming model

First, the necessity measure, that is applied to defuzzify the fuzzy numbers, is explained. If the uncertain model parameters are presumed based on the chance and trapezoid fuzzy numbers denoted by $\omega =\left(\omega_{1},\omega_{2},\omega_{3},\omega_{4}\right)$, the equivalent crisp number is obtained using Formula (36):

(36) $$E\left(\omega \right)=\frac{\omega_{1}+\omega_{2}+\omega_{3}+\omega_{4}}{4}$$

The necessity of the constraint $\hat{\omega }\leq r$ is defined in Formula (37):

(37) $$Nec\left(\hat{\omega }\leq r\right)=\left\{ \begin{array}{c} 1\;\omega_{4}\leq r\;\\ \;\\ \frac{r-\omega_{3}}{\omega_{4}-\omega_{3}}\;{\;\omega }_{3}\leq r\leq \omega_{4}\;\\ 0\;\omega_{3}\geq r\; \end{array}\right.$$

Nec denotes the necessity measure in the chance-based constraint programming.

Based on the upper formula, if α≥0.5:

(38) $$Nec\left(\hat{\omega }\leq r\right)\geq \alpha \;↔\;r\geq \left(1-\alpha \right)\times \omega_{3}+\alpha \times \omega_{4}$$

Relation (38) can be directly used to convert the fuzzy constraints into their equivalent crisp constraints.

**Fig. 3:**
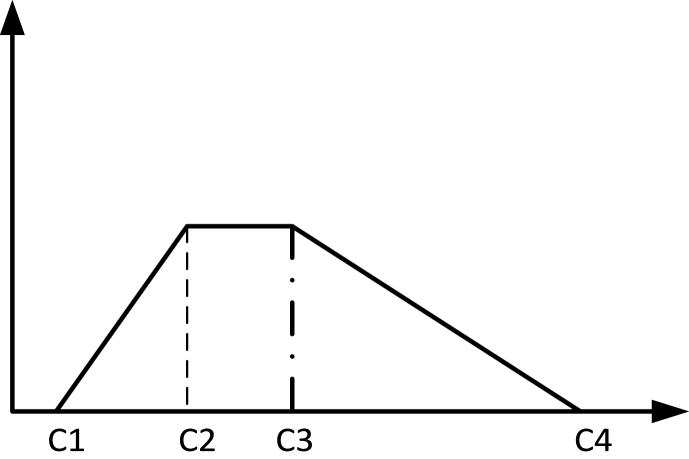
Fuzzy parameter with trapezoidal distribution.

In this study, the trapezoidal fuzzy numbers are employed for the fixed and variable costs, shown in Fig. 3:

According to Formula (36) and Relation (38), the uncertain mathematical programming model presented in the previous section can be defuzzified shown in Equation (39) and Constraints (40–42):

$$Minimize\;Z_{1}=E\left(Z_{1}\right)=\sum_{i\in N_{p}\cup N_{d}}\sum_{j\in N_{p}\cup N_{d},\left(i\neq j\right)}\sum_{v\in V}c_{ij}s_{ij}^{v}+\sum_{\frac{i\varepsilon N}{N_{p}}}\sum_{j\in N_{c}}c_{ij}x_{ij}$$

(39) $$+\sum_{k\in N_{d}}E\left[{FD}_{k}\right]y_{k}+\sum_{i\in N_{p}}\sum_{j\in N_{d}}\sum_{v\in V}{E[FV}_{1}^{v}]s_{ij}^{v}+\sum_{i\varepsilon N_{c}}\sum_{k\in N_{d}}{E[FV}_{2}]x_{ki}$$

(40) $$Nec\left\{\sum_{j\in N_{d}}\sum_{v\in V}Q_{ij}^{v}\leq {CP}_{i}\right\}\geq \alpha \forall i\in N_{p}$$

(41) $$Nec\left\{\sum_{i\in N_{c}}{d_{i}w}_{ik}\leq {CD}_{k}\right\}\geq \beta \forall k\in N_{d}$$

(42) $$Nec\left\{\sum_{i\in N_{c}}x_{ki}\leq NV\right\}\geq \gamma \forall k\in N_{d}$$

Here, the converted parts corresponding to the first objective function and the constraints of the primary model that have fuzzy numbers are presented. The robust model considering feasibility robustness and optimality robustness is expressed using Equation (43) and Constraints (44–46):

$$Minimize\;Z_{1}=E\left(Z_{1}\right)=\sigma \times \left(Z_{1\;\mathrm{Max}}-Z_{1\;\mathrm{Min}}\right)$$

$$+\sum_{i\in N_{p}\cup N_{d}}\sum_{j\in N_{p}\cup N_{d},\left(i\neq j\right)}\sum_{v\in V}c_{ij}s_{ij}^{v}+\sum_{\frac{i\varepsilon N}{N_{p}}}\sum_{j\in N_{c}}c_{ij}x_{ij}$$

$$+\sum_{k\in N_{d}}\frac{{FD}_{k}^{1}+{FD}_{k}^{2}+{FD}_{k}^{3}+{FD}_{k}^{4}}{4}y_{k}+\sum_{i\in N_{p}}\sum_{j\in N_{d}}\sum_{v\in V}\frac{{FV}_{11}^{v}+{FV}_{12}^{v}+{FV}_{13}^{v}+{FV}_{14}^{v}}{4}s_{ij}^{v}+\sum_{i\varepsilon N_{c}}\sum_{k\in N_{d}}\frac{{FV}_{2}^{1}+{FV}_{2}^{2}+{FV}_{2}^{3}+{FV}_{2}^{4}}{4}x_{ki}$$

$$+\delta 1\times \left(\sum_{i\in N_{p}}{\alpha \times CP}_{i}^{1}+{\left(1-\alpha \right)\times CP}_{i}^{2}-{CP}_{i}^{1}\right)$$

$$+\delta 2\times \left(\sum_{k\in N_{d}}{\beta \times CD}_{k}^{1}+\left(1-{\beta )\times CD}_{k}^{2}-{CD}_{k}^{1}\right)\right)$$

(43) +δ3×(γ×NV1+(1−γ)×NV2−NV1)

(44) $$\sum_{j\in N_{d}}\sum_{v\in V}Q_{ij}^{v}\leq {\alpha \times CP}_{i}^{1}+{\left(1-\alpha \right)\times CP}_{i}^{2}\forall i\in N_{p}$$

(45) $$\sum_{i\in N_{c}}{d_{i}w}_{ik}\leq {\beta \times CD}_{k}^{1}+(1-{\beta )\times CD}_{k}^{2}\forall k\in N_{d}$$

(46) $$\sum_{i\in N_{c}}x_{ki}\leq \gamma \times NV1+\left(1-\gamma \right)\times NV2\forall k\in N_{d}$$

In the above model, the minimum confidence level of the fuzzy constraints (α, β, and γ) must be determined by the decision-maker in such a way that like the sensitivity analysis method, the amounts of the parameters must be changed. As the number of fuzzy constraints increases, the number of tests required to determine the appropriate values of confidence levels increases significantly. Also, the model is not sensitive to the deviation of the objective function value from its optimal value, and this can impose a high risk on the decision-maker in real-world problems. Therefore, the application of the robust possibilistic approach is effective to minimize the imposed risk [55].

The first expression $E\left(Z_{1}\right)$ represents the expected value of the first objective function. The second expression, $\sigma \times \left(Z_{1\;\mathrm{Max}}-Z_{1\;\mathrm{Min}}\right)\text{,}$ represents the difference between the two boundary values of $Z_{1\;\mathrm{Max}}$ and $Z_{1\;\mathrm{Min}}$, which are obtained by placing the upper bound amounts and the lower bound amounts of the parameters in the objective function $Z_{1}$, respectively. σ indicates the importance of this expression compared to other expressions of the objective function. Therefore, the second expression results in minimizing the maximum negative and positive deviations from the expected optimal value of $Z_{1}$. In addition, this expression controls the optimality robustness of the solution vector. The subsequent expressions specify the confidence level associated with every fuzzy constraint in which $\delta_{i}$ is the possible deviation penalty of constraint *i* that contains the uncertain parameter. The last three expressions of the first objective function determine the difference between the value used in the chance-based constraint with the best value of the uncertain parameter. In addition, these last three expressions control the feasibility robustness.

## 4 Solving methodology

In this study, the Augmented Epsilon Constraint (AEC) method is used to solve the multi-objective optimization model and obtain the efficient Pareto frontier. Due to the NP-Hardness of the location-routing problem, two metaheuristic algorithms named NSGA-II and MOSFS are employed to deal with this problem.

### 4.1 The Augmented Epsilon Constraint (AEC) method

In the AEC method, first the appropriate range for changing the objective function values $\left(e_{i}\right)$ must be determined and then the Pareto front is obtained for different values of $\left(e_{i}\right)$. For better implementation of this method, the appropriate ranges of epsilons $\left(e_{i}\right)$ should be determined using the Lex method. The two main steps in this method contain: determining the range of the $e_{i}$ values, and solving the AEC model. The AEC model of this research is presented as Model (47) [56].

(47) $$\left\{ \begin{array}{c} \mathrm{Min}\;OB_{1}\left(y\right)-\sum_{i=2}^{n}{\beta_{i}s}_{i}\;\\ {OB}_{i}\left(y\right)+s_{i}={epsilon}_{i}\;i=2,3,..,I\\ y\in Y\;\\ s_{i}\geq 0\; \end{array}\right.$$

where $s_{i}$ are nonnegative slack variables, and $\beta_{i}$ is a parameter for normalizing the value of the first objective function in relation to the goal *i*: $\beta_{i}=\frac{R\left({OB}_{1}\right)}{R\left({OB}_{i}\right)}$. To solve the single-objective model, the cost objective function is defined as the main goal and the two other objective functions are bounded: ${epsilon}_{i}\in \left[\mathrm{Min}\left({OB}_{i}\right),\mathrm{Max}\left({OB}_{i}\right)\right]$*.* The obtained solution is efficient and is placed on the Pareto frontier. Note that by changing the $e_{i}$ values, another efficient solution and its corresponding point on the Pareto frontier is obtained.

### 4.2 Initial solution representation method

The continuous solution representation is employed for this problem. The solution representation contains a string of decimal numbers ranging from 0 to 1 with the length of Nc+Nd−1+V+Nd+V−1. V represents the set of available vehicles in each facility, Nd denotes the second-echelon facility, and Nc denotes the total number of customers at the second echelon. For instance, a solution representation for a numerical example with V=2,Nd=3,Nc=5 can be shown as follows:

**Table:** 

0.35	0.82	0.01	0.04	0.16	0.64	0.73	0.64	0.45	0.54	0.29	0.74	0.18

The first section of the solution representation with Nc+Nd−1 dimensions is corresponding to routing from depot to customers.

**Table:** 

0.35	0.82	0.01	0.04	0.16	0.64	0.73

The permutation of the numbers of this part is obtained by arranging in a descending order.

**Table:** 

3	4	5	1	6	7	2

In this permutation, the numbers that are greater than Nc are considered as the separators, so that any series of numbers that are less than Vc , which are placed in sequence, corresponding with a route starting from a facility and ending at the last point of that group. In this example, the formed route initiates from the first facility and finishes after passing the route of 3−4−5−1.

The second part of the solution representation with the length V is corresponding to the allocation of the vehicles to the factories.

**Table:** 

0.64	0.45

Decimal numbers are assigned to one of the factories according to the following formula. Np represents the number of factories:

⌊x*Np+1⌋

**Table:** 

2	2

The third section of the solution representation with the length of *Nd + V -1* is corresponding to routing from factories to facilities.

**Table:** 

0.54	0.29	0.74	0.18

Same as the first part, the routes linking factories and facilities are found after sorting in descending order using separators.

**Table:** 

4	2	3	1

In this example, the route that can be formed starts from the second factory and passes through 2-3-1. It should be noted that facilities that are not used will be removed from this route. In this example, as only the first facility is utilized, the route originates from the second factory to the first facility and vice versa. So far, the factories and routes have been determined. Then, according to the route, the number of commodities and goods loaded at the starting of the route, the number of goods unloaded at each node and the traveled length can be calculated.

### 4.3 Non-dominated Sorting Genetic Algorithm (NSGA-II)

Genetic algorithm (GA) is one of the well-known metaheuristic algorithms that has emerged from biological models of living organisms. In this algorithm, the natural selection process is simulated so that the fittest and most desirable solutions are chosen to produce offspring of the next generation. The NSGA-II algorithm is a multi-objective GA that contains the following steps [57,58]:

(1) Creating an initial population
(2) Calculating the fitness criteria
(3) Non-dominated sorting the population and calculating crowding distance Performing crossover and mutation operations to produce new offspring
(4) Combining the initial population and the population created by the crossover and mutation operations
(5) Replacing the initial (parent) population with the fittest (the most desirable) members of the combined population created in the previous step
(6) All steps are repeated until the desirable generation (or optimal solutions) are achieved.

#### 4.3.1 Crossover operator (One-point crossover)

**Fig. 4:**
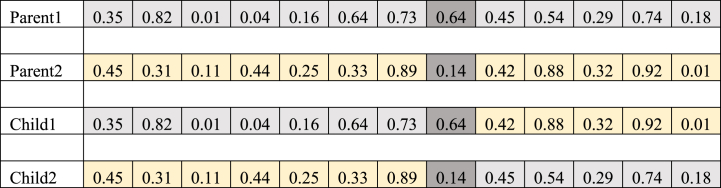
A crossover representation for the chromosome of the sample problem.

In the first step, it randomly selects a pair of chromosomes (solutions). In the second step, it randomly selects a location along the chromosome string, and finally in the third step, it swaps the amounts of the two strings regarding the location selected in the previous step. It should be noted that the crossover rate must be adjusted appropriately to determine what percentage of the current population is selected for crossover operation. An example of the crossover operation is represented in Fig. 4.

#### 4.3.2 Mutation operator

**Fig. 5:**
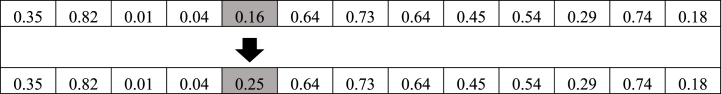
A mutation representation for the chromosome of the sample problem.

This operation is usually applied to a single chromosome (solution), which causes some of the chromosome genes to be randomly changed to produce a new offspring. This operator increases the scatter of solutions and reduces the probability of getting stuck in a local optimum. In this research, a gene is randomly selected and a new amount is randomly assigned to that given gene. An example of the mutation operation is shown in Fig. 5.

### 4.4 Multi-Objective Stochastic Fractal Search (MOSFS)

The Stochastic Fractal Search (SFS) algorithm is an efficient metaheuristic algorithm introduced by Salimi [59]. This algorithm has been used in various research topics. The Multi-Objective version of this algorithm (MOSFS) is able to solve complex multi-objective optimization problems by obtaining an efficacious set of the best non-dominated solutions with increased diversity. This algorithm is also capable of efficiently searching the solution space. Moreover, exploration and exploitation features are ensured by systematic random walks together with adaptive jump distance. Furthermore, the MOSFS algorithms contains fewer parameters to be tuned. For more information about the characteristics and advantages of the MOSFS algorithms, please refer to Ref. [60].

### 4.5 Problem design

A set of Sterle standard problems are used to test and evaluate the proposed model [61]. These problems are known according to the location of facilities and customers in three groups including I1, I2 and I3, and each group is known by 31 problems, represented in Table 1.

**Table 1:** Specifications of Sterle standard problems.

Instance	Sterle [61]
Instance Format	No. of Customers-No. of Potential Depots-No. of Plants
No. of instances	93 Total (31 each of I1, I2 and I3)
No. of Plants	{2, 3, 4, 5}
No. of Depots	{3, 4, 5, 8, 10, 15, 20}
Depots Location Costs	Linear function of the capacity values and vary in the range [40,80]
No. of Customers	{8, 9, 10, 12, 15, 20, 25, 50, 75, 100, 150, 200}
1^st^ level vehicles capacity/2^nd^ level vehicle capacity	{300, 500, 800}/200 (Up to 21 Nodes) {500, 800, 1000}/200 (Up to 36 Nodes) {800, 1000, 1600}/200 (Up to 39 Nodes) {1500, 2500, 3500}/200 (Up to 125 Nodes) {3000, 5000, 8000}/500 (Up to 225 Nodes)
Customer location	Randomly distributed
Customer demand	Randomly generated in the range [1,100]
$(\omega_{em}=\left[0.5\right],\omega_{ed}=\left[0.5\right],{JO}_{k}=\left[50,100\right],{EV}_{k}=\left[0.5,0.100\right],{ur}_{k}=\left[0.1,1\right],{rd}_{k}=\left[0.1,1\right]$	

In this paper, the group of I2 problems with the following specifications is considered, shown in Table 2.

**Table 2:** Design of instance problems for comparing the performance of algorithms.

Problem	Plant	Depot	Costumer	Problem	Plant	Depot	Costumer
1	2	3	8	16	4	10	20
2	2	4	8	17	2	8	25
3	2	3	9	18	3	8	25
4	2	4	10	19	2	10	25
5	3	5	10	20	3	10	25
6	3	8	10	21	4	10	25
7	2	4	15	22	5	8	50
8	3	5	15	23	5	10	50
9	3	8	15	24	5	10	75
10	2	10	15	25	5	15	75
11	3	10	15	26	5	10	100
12	2	8	20	27	5	20	100
13	3	8	20	28	5	10	150
14	2	10	20	29	5	20	150
15	3	10	20	30	5	10	160
				31	5	15	160

### 4.6 Adjusting the parameters of the algorithms using the Taguchi method

The performance of any metaheuristic algorithm is strongly affected by the amount set for its parameters.

#### 4.6.1 Parameter setting for the NSGA-II and MOSFS algorithms

Four NSGA-II parameters including *MaxIt* (maximum iteration), *NPOP* (number of initial population), *PC* (crossover rate), and *PM* (mutation rate) must be set at their optimal levels. Three MOSFS parameters including *MaxIt* (maximum iteration), Diff (number of initial population), and Walk (harmonic memory coefficient) must be set at their optimal levels. In this section, three levels are considered for each parameter. For this purpose, problem number 15, which encompasses 3 factories, 10 depots, and 20 customers, is chosen. For each parameter, three levels of low (1), medium (2) and high (3) are defined separately to solve the problem, shown in Table 3.

**Table 3:** Different levels of the NSGA-II parameters.

Parameter	Level		
	1	2	3
MaxIt	150	250	350
NPOP	100	150	200
PC	0.7	0.75	0.8
PM	0.25	0.3	0.35
Walk	1	0.25	0
Diff	1	5	9

After determining the levels for the algorithm parameters using the Taguchi method and the MINITAB software, the required experiments are designed, shown in Table 4. It should be noted that each experiment is performed 10 times and their average is recorded to reduce the experimental errors

**Table 4:** Experiments designed by the Taguchi method for NSGA-II and MOSFS.

	NSGA_II				MOSFS	
Number of experiment	Max iterations	Population size	*PC*	*PM*	Walk	Diff
1	1	1	1	1	1	1
2	1	2	2	2	1	2
3	1	3	3	3	1	3
4	2	1	2	3	2	1
5	2	2	3	1	2	2
6	2	3	1	2	2	3
7	3	1	3	2	3	1
8	3	2	1	3	3	2
9	3	3	2	1	3	3

The indices required for setting the parameters include SNS, MID, Diversity, Spacing, and RAS, which are explained as follows.

### 4.7 The evaluation metrics

Six indices have been proposed in the literature so far for the performance analysis of the multi-objective optimization algorithms [62] These indices are explained as follows.

#### 4.7.1 Time

The less run time the better performance of an algorithm.

#### 4.7.2 MID

The mean of deviations of the Pareto solutions from the ideal point $I_{sol}$ = $\min\left(z_{1},z_{2}\right)$ (in which the values of both objective functions are optimal or the origin of the coordinates can be considered as the ideal point for the minimization problems) is measured by MID (Mean Ideal Distance) index [63]. For computing the MID value of maximizing objective function, the values of Pareto solutions are become inverse and the ideal point is considered (0). The value of MID is calculated using Equation (48).

(48) $$MID\left(A\right)=\frac{\sum_{PA\in F\left(A\right)}{∥pa-I_{sol}∥}_{2}}{∥F\left(A\right)∥}$$

where ${∥pa-I_{sol}∥}_{2}$ denotes the Euclidean distance between paℇF(A) and the ideal point. The lower MID value the better performance of an algorithm.

#### 4.7.3 Diversity

The Diversity index, which is computed using Equation (49), measures the diameter of a space cube used by the end values of objective functions for a set of non-dominated solutions. The more Diversity value the better [64].

(49) $$DM=\sqrt{{\left({maxf}_{1i}-\min\;f_{1i}\right)}^{2}+{\left({maxf}_{2i}-\min\;f_{2i}\right)}^{2}}$$

#### 4.7.4 Spacing (S)

The Spacing index calculates the relative distance of successive solutions using Equation (50). The smaller Spacing index the better.

(50) $$S={\left(\frac{1}{n_{pf}}\sum_{i=1}^{n_{pf}}{\left(d_{i}-\bar{d}\right)}^{2}\right)}^{\frac{1}{2}},where\;\bar{d}=\frac{1}{n_{pf}}\sum_{i=1}^{n_{pf}}d_{i\;}$$

#### 4.7.5 RAS

The value of the RAS (Rate of Achievement to two objectives Simultaneously) is calculated using Equation (51) [65].

(51) $$RAS=\frac{\sum_{i=1}^{n}\left(\frac{f_{1i}-F_{i}}{F_{i}}\right)+\left(\frac{f_{2i}-F_{i}}{F_{i}}\right)}{n}$$

in which, $F_{i}=\min\left\{f_{1i},f_{2i}\right\}$. The smaller RAS value the better.

#### 4.7.6 SNS

The value of SNS (Spread of Non-dominance Solutions) is calculated by Equation (52) [65].

(52) $$SNS=\sqrt{\frac{\sum_{i=1}^{n}{\left(MID-c_{i}\right)}^{2}}{n-1}}$$

The higher SNS values the more diversity of solutions, in other words, the better solution quality.

After ten runs, the average is reported for each test result, presented in Table 5.

**Table 5:** The outputs of algorithm for indices.

Diversity	Spacing	MID	SNS	RAS
514.351	239.6004	685.5478	163.6435	0.61473
465.9142	304.8409	654.2415	151.5697	0.62842
465.9142	221.7025	646.5023	146.4427	0.62842
472.5177	262.6944	686.9998	161.6445	0.56401
482.5536	229.7129	712.7831	146.9922	0.43207
465.9142	218.9298	675.0498	136.1148	0.47131
465.9142	193.3478	601.016	161.1326	0.94262
465.9142	304.8409	654.2415	151.5697	0.62842
465.9142	218.9298	675.0498	136.1148	0.47131

In order to obtain an output for each experiment, all the indices are normalized using Equations (53), (54), represented in Table 6.

(53) $$x_{j}^{+}\to R_{ij}=\frac{r_{ij}-\min\left(r_{ij}\right)}{\max\left(r_{ij}\right)-\min\left(r_{ij}\right)}$$

(54) $$x_{j}^{-}\to R_{ij}=\frac{\max\left(r_{ij}\right)-r_{ij}}{\max\left(r_{ij}\right)-\min\left(r_{ij}\right)}$$

**Table 6:** Normalized indices.

Diversity	Spacing	MID	SNS	RAS
1	0.58515	0.24368	1	0.64223
0	0	0.52378	0.56141	0.61541
0	0.74568	0.59303	0.37517	0.61541
0.13633	0.37802	0.23069	0.92738	0.74157
0.34353	0.67384	0	0.39513	1
0	0.77055	0.33761	0	0.92314
0	1	1	0.90879	0
0	0	0.52378	0.56141	0.61541
0	0.77055	0.33761	0	0.92314

In this normalization method, the indices with a negative nature are converted into the positive indices. The indices are prioritized using Goal Programming based on their importance and a weight is considered for each index accordingly [64,65]. Subsequently, the total weights of the indices for each experiment are calculated using Equation (55) according to the importance weights.

(55) $$Response=\sqrt[{2}]{{\left(MID\right)}^{2}+{\left(RAS\right)}^{2}+{\left(Spacing\right)}^{1}+{\left(SNS\right)}^{1}+{\left(SNS\right)}^{1}{+\left(Diversity\right)}^{1}}$$

**Fig. 6:**
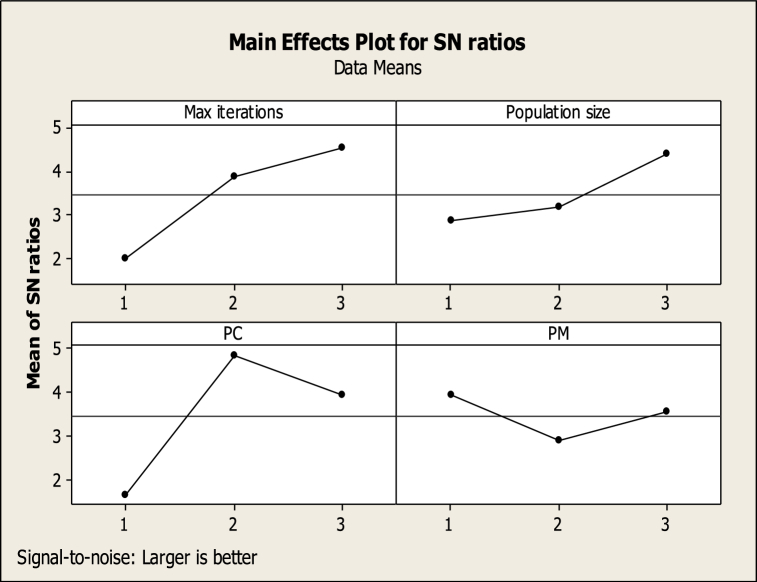
The output of the MINITAB software (the NSGA-II parameters).

**Fig. 7:**
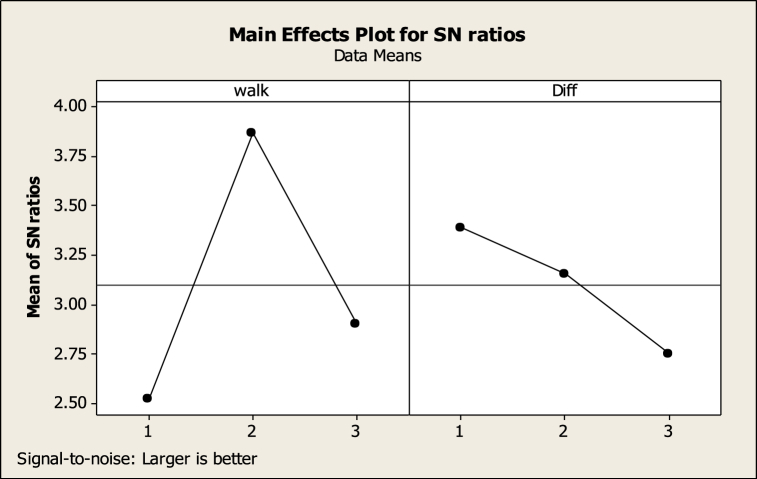
The output of the MINITAB software (the MOSFS parameters.

According to the Response values, $\frac{S}{N}$ is computed and the parameters’ levels are determined. The MINITAB outputs are depicted in Fig. 6, Fig. 7.

The levels of the NSGA-II and MOSFS parameters are represented in Table 7.

**Table 7:** The NSGA-II and MOSFS parameters.

Max iterations	Population size	PC	PM	Walk	Diff
**350**	200	0.75	0.25	0.25	1

## 5 Computational results

The proposed model was coded in a notebook system with Intel Core™ i5 processor, 4 Gb RAM, and Microsoft Windows 10 Ultimate operating system. First, the AEC method as an exact method was used through the GAMS optimization software to validate the proposed model. Then, the sensitivity analysis was performed to investigate the effects of changing the main parameters of the model on the optimal values of the objective functions. Subsequently, the NSGA-II and MOSFS metaheuristic algorithms were employed through MATLAB version 2018a software for solving the large size problems. Finally, the performance of NSGA-II and MOSFS were compared in terms of six different indexes.

### 5.1 Model validation

An instance problem with 20 nodes, in which spatial coordinates were specified and Euclidean distances were considered, was designed and solved using the AEC method together with two NSGAII and MOSFS metaheuristic algorithms. At the first level of this problem, there are three factories with capacities of 15,000, 15,000 and 30,000 units. At the second level, there exist different five potential locations for setting up depots with the same capacity of 8000 units. Also, the set-up cost of each depot is 150. In addition, there are 12 customers with deterministic and known demands. The transport fleet of the first echelon is limited and heterogeneous, and each factory has three vehicles with different types. The capacities of these vehicles are 7000, 14000, and 20,000 units, respectively, and the usage fixed costs of vehicles are 30, 20, and 10 monetary units, respectively. The transport fleet of the second echelon is homogenous and unlimited. The capacity of these vehicles is the same and equal to 5000 units, also, the usage fixed cost of each vehicle is 100 monetary units. The carbon dioxide emissions of all vehicles are taken from: “Environmental Reporting Guidelines: Including streamlined energy and carbon reporting guidance_March 2019” (conversion factors 2021). The parameters of the first objective function are considered as follows:

$$\omega_{em}=0.5,\omega_{ed}=0.5,{JO}_{k}=50-100,{EV}_{k}=0.5-0.100,{ur}_{k}=0.1-1,{rd}_{k}=0.1-1\text{.}$$

**Fig. 8:**
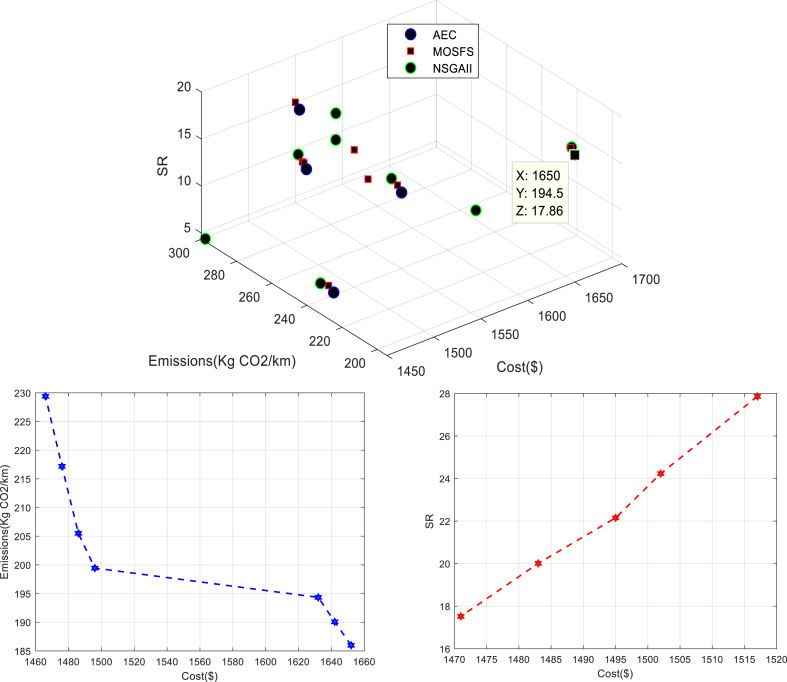
Pareto frontiers obtained by using the AEC, NSGA-II, and MOSFS.

The Pareto frontiers obtained by using the AEC, NSGA-II, and MOSFS are depicted in Fig. 8.

**Fig. 9:**
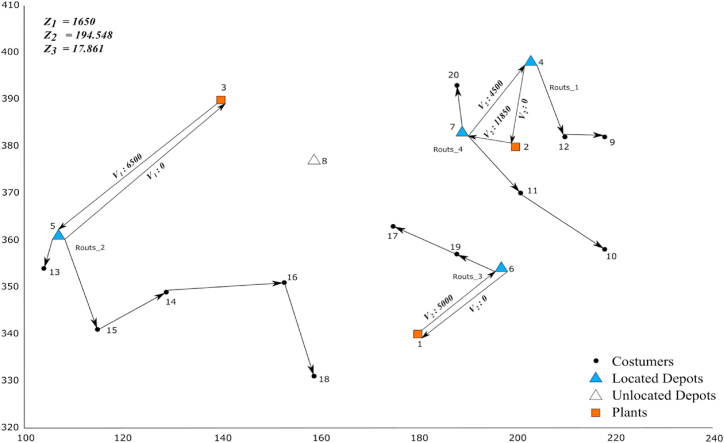
Solution representation.

The Pareto frontiers obtained by AEC, NSGA-II, and MOSFS are presented in Fig. 9.

As shown in Fig. 9, the established depots and the created routes in the first and second echelons are plotted. depots are located in four locations out of the five potential locations, and routes are originated from depots to deliver goods to customers (6 trips are started from 4 depots; two trips are started from each of depots 5 and 7 and one trip is started from each of depots 6 and 4). Taking into account the fixed cost of set-up depots as well as the fixed cost of using vehicles in the first and second echelon, the total cost is equal to 1650 monetary units, which is the highest total cost among all of the obtained Pareto solutions. Regarding the second objective function, the model chooses more vehicles with less environmental pollution so that the number of trips in both echelons is increased and the distance traveled per trip is reduced which results in reducing CO_2_ emissions to the lowest level of 194.548 among all of the obtained Pareto solutions. With regard to the third objective function, the employment and economic development rates rise as a result of setting up four depots which lead to rising the third objective function value to the maximum value of 17.861 among all of the obtained Pareto solutions.

### 5.2 Sensitivity analysis

**Fig. 10:**
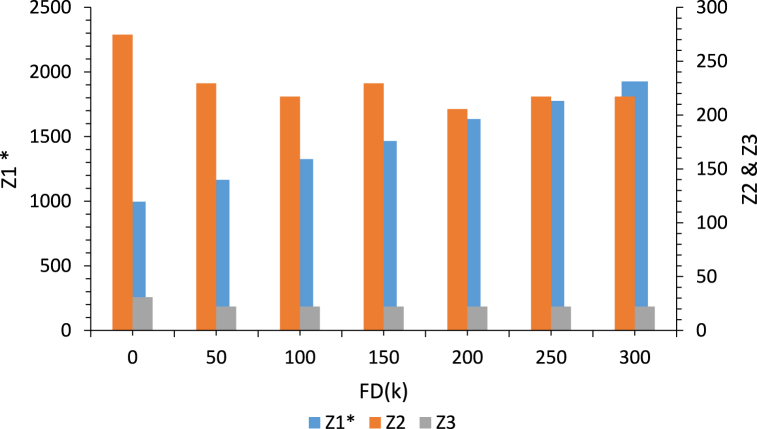
Analysis of the effect of changes in the depots set-up cost $\left({FD}_{k}\right)$ on the values of three objective functions.

The effect of the changes in the fixed cost of set-up depots $\left({FD}_{k}\right)$ on the values of three objective functions is depicted in Fig. 10. In this scenario, the first objective function value $\left(Z_{1}\right)$ is considered optimal and the other model parameters are fixed.

As can be seen in Fig. 10, increasing the amount of depots set-up cost leads to increasing the value of the first objective function $\left(Z_{1}\right)$ and the value of the second objective function $\left(Z_{2}\right)$. However, the value of the third objective function $\left(Z_{3}\right)$ is decreased. Because, if we set up a number of depots, the total costs of the entire system will be increased. Also, the regional development and employment rates will be increased. However, the total amount of CO_2_ emissions will be decreased.

**Fig. 11:**
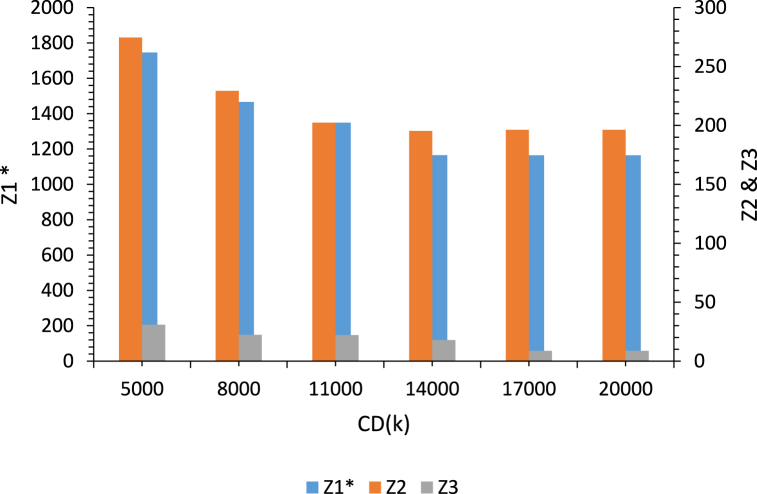
Analysis of the effect of changes in the capacity of depots $\left({CD}_{k}\right)$ on the values of three objective functions.

The effect of changes in the capacity of depots $\left({CD}_{k}\right)$ on the values of three objective functions is illustrated in Fig. 11. In this scenario, the first objective function value $\left(Z_{1}\right)$ is also optimal and the other model parameters are fixed.

As shown in Fig. 11, increasing the capacity of the depots leads to decreasing the values of the three objective functions $\left(Z_{1}{,Z}_{2},Z_{3}\right)$. Because, increasing the capacity of the depots results in decreasing the number of established depots that means decreasing the depots set-up costs and transportation costs. On the other hand, increasing the capacity of the depots leads to decreasing the total amount of CO_2_ emissions, because the distance traveled by vehicles is reduced. Eventually, increasing the capacity of the depots results in decreasing the value of the third objective function, which is related to the social responsibility, as increasing the capacity of depots means decreasing the number of established depots and reducing the regional development and employment rates.

### 5.3 Comparison of the performance of the NSGA-II and MOSFS algorithms

Subsequently, the group of I2 problems 31 problems (the specifications of the problems are presented in Table 1) introduced by Sterle [61] were used to compare the performance of two metaheuristic algorithms. For each metaheuristic algorithm, every problem was run ten times and the average was reported as the final solution, the values of all the indices are shown in Table 8.

**Table 8:** The outputs of NSGA-II and MOSFS.

NSGAII							MOSFS					
	Time	Diversity	Spacing	MID	SNS	RAS	Time	Diversity	Spacing	MID	SNS	RAS
1	8.64	480.71	20.71	688.1	128.11	0.289	6.65	480.71	20.71	688.14	128.11	0.289
2	20.22	469.93	197.45	682.3	130.04	0.47	15.55	469.93	197.45	682.3	130.04	0.47
3	49.15	630.67	41.34	718.8	155.34	0.436	37.81	630.67	41.34	718.78	155.34	0.436
4	104.40	483.1	37.2	809.6	150.93	0.556	80.31	476.37	37.2	811.81	148.69	0.539
5	155.83	427.55	59.24	693.8	116.4	0.573	119.87	380.27	59.24	709.91	100.31	0.436
6	249.86	334.44	107.39	672.4	45.76	0.255	192.20	334.44	107.39	672.35	45.76	0.255
7	380.05	492.08	37.1	770.4	125.61	0.269	292.35	492.08	37.1	770.44	125.61	0.269
8	606.11	598.96	15.69	686.6	189.13	0.293	466.24	719.91	46.85	705.97	211.52	0.358
9	711.63	574.72	27.61	718.4	146.86	0.374	547.41	574.72	27.61	718.41	146.86	0.374
10	953.49	441.62	44.84	686.1	93.72	0.128	733.45	435.36	40.34	697.88	105.36	0.154
AV_S	323.94	493.378	58.857	712.65	128.19	0.3643	249.18	499.446	61.523	717.599	129.76	0.358
11	1325.70	500.37	38.25	582.9	112.18	0.333	946.93	500.37	38.25	582.88	112.18	0.333
12	1755.71	375.56	4.04	607.6	105.32	0.314	1254.08	353.16	233.25	569.2	94.34	0.392
13	2130.07	696.97	40.64	744.2	209.16	0.38	1521.48	683.85	21.42	681.59	187.85	0.214
14	2790.30	411.17	22.56	589	120.59	0.188	1993.07	590.12	28.43	655.64	164.75	0.213
15	3073.17	576.4	16.9	699.6	158.82	0.188	2195.12	594.25	14.7	675.41	166.76	0.188
16	3565.32	446.43	155.6	556.6	70.48	0.199	2546.66	446.43	155.6	556.61	70.48	0.199
17	4988.76	479.42	12.93	629	154.46	0.242	3563.40	520.31	22.81	570.58	148.94	0.248
18	5791.34	564.88	178.99	587.8	114.61	0.205	4136.67	677.86	143.19	529.04	154.46	0.185
19	6749.13	533.65	58.93	562.5	112.07	0.341	4820.81	640.38	47.14	506.28	123.28	0.306
20	7706.92	742.3	53.97	757.1	209.13	0.453	5504.94	742.3	43.17	681.38	230.05	0.407
AV_M	3987.64	532.715	58.281	631.63	136.682	0.2843	2848.32	574.903	74.796	600.861	145.31	0.2685
21	9248.30	750.555	87.975	757.77	201.92	0.4329	7156.42	850.629	68.85	641.168	258.01	0.3663
22	10305.25	563.34	9.292	789.88	189.58	0.4082	8587.71	600.372	419.85	626.12	216.98	0.4312
23	11164.02	1045.46	93.472	967.46	376.49	0.494	9303.35	1162.55	38.556	749.749	432.06	0.2354
24	13396.82	616.755	51.888	765.7	217.06	0.2444	11164.02	1003.2	51.174	721.204	378.93	0.2343
25	14513.22	864.6	38.87	909.48	285.88	0.2444	12094.35	1010.23	26.46	742.951	383.55	0.2068
26	17415.87	669.645	357.88	723.58	126.86	0.2587	14513.22	758.931	280.08	612.271	162.1	0.2189
27	18867.19	719.13	29.739	817.7	278.03	0.3146	15722.66	884.527	41.058	627.638	342.56	0.2728
28	22640.63	847.32	411.68	764.14	206.3	0.2665	18867.19	1152.36	257.742	581.944	355.26	0.2035
29	24527.35	800.475	135.54	731.25	201.73	0.4433	20439.46	1088.65	84.852	556.908	283.54	0.3366
30	29432.82	1113.45	124.13	984.23	376.43	0.5889	24527.35	1261.91	77.706	749.518	529.12	0.4477
31	31885.55	1426.43	112.72	1237.2	551.14	0.7345	26571.29	1435.17	70.56	942.128	774.69	0.5588
AV_L	18490.64	856.105	132.11	858.95	273.77	0.40276	15358.82	1018.96	128.808	686.509	374.25	0.3193
AV_T	7600.74	627.399	83.082	734.41	179.55	0.35045	6152.11	697.769	88.3757	668.323	216.44	0.3153

**Fig. 12:**
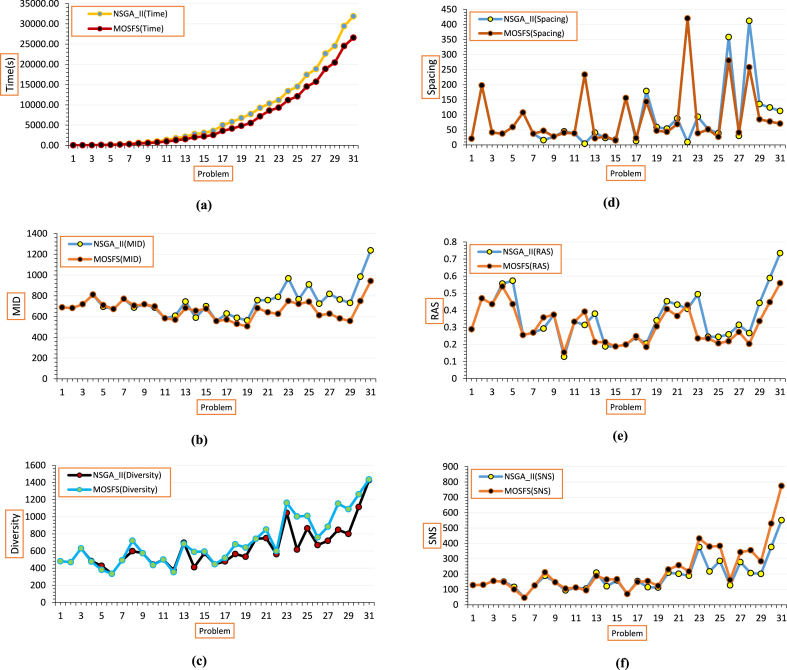
**(a).** Comparing the Run time of NSGA-II and MOSFS, **(b).** Comparing the MID value of NSGA-II and MOSFS, **(c).** Comparing the Diversity value of NSGA-II and MOSFS, **(d).** Comparing the Spacing value of NSGA-II and MOSFS, **(e).** Comparing the RAS value of NSGA-II and MOSFS, **(f).** Comparing the SNS value of NSGA-II and MOSFS.

The comparison of the performance of two NSGA-II and MOSFS metaheuristic algorithms is illustrated in Fig. 10 in terms of Time, MID, Diversity, Spacing, RAS, SNS. As shown in Fig. 12 (a), the solution time of MOSFS is less than the solution time of NSGAII. As a result, MOSFS algorithm performs better according to Time. The less MID value, the better performance of the algorithm. Fig. 12(b) indicates that MOSFS outperforms NSGA-II based on the MID index. The more Diversity value the better. Therefore, the MOSFS algorithm performs better in terms of this measure, displayed in Fig. 12(c). As can be seen in Fig. 12(d), in some problems, MOSFS performs better in terms of Spacing, but, NSGA-II outperforms in some other problems. The less RAS value the better. According to Fig. 12(e), the MOSFS algorithm has a better performance based on the RAS index. The more SNS value the better. As can be seen in Fig. 12(f), there is not considerable difference between the two algorithms for small and medium size problems, however the performance of the MOSFS algorithm is better for the larger problems.

It can be concluded from the above figures that there is no considerable difference in the performance of two algorithms for the small size problems. However, as the problem size grows, this difference rises significantly and the MOSFS algorithm performed better. For example, based on the Time index, as the problem size increases, the computational time for both algorithms increases, but the run time of the MOSFS algorithm increases with a slighter slope than the NSGAII algorithm. As can be seen in Table 8, for the small-sized problems, the NSGA-II algorithm outperforms the MOSFS algorithm in terms of Time, Diversity, RAS, and SNS indices. For the medium-sized problems, NSGA-II outperforms based on Spacing, and MOSFS performs better in terms of the other five indices. Finally, for the large-sized problems, the MOSFS algorithm outperforms in terms of all six indices. In general, NSGAII outperforms only according to Spacing and MOSFS outperforms based on Time, RAS, Diversity, MID, and SNS.

## 6 Conclusion

During the decades, governments have paid a lot of attention the increase of economic growth and employment rate. On the other hand, economic and population growth, and technological advancement have had significant detrimental impacts on the environment and natural resources. Therefore, sustainable development based on three main aspects including economy, environment, and social responsibility plays a crucial role in the performance of supply chains. Nowadays, many transportation operations of the supply chains are performed by logistic companies. The concept of open routing has been introduced due to the limited number of available vehicles in a logistic company as a percentage of transportation activities are performed by a logistic firm.

For this purpose, in this paper, both open and closed routes were taken into account in the sustainable 2E-LRP. The hybrid closed and open routes in a sustainable 2E-LRP can be mentioned as the main contribution of the proposed model. A multi-objective mixed integer linear programming (MILP) model including minimization of costs and CO_2_ emissions and maximization of social responsibility (creating job opportunities and community development) was developed for the problem in hand. It should be noted that optimizing the three objectives of the problem is not possible as these objectives are in conflict with each other. The proposed model seeks to minimize the total costs by reducing the use of vehicles through reducing the number of transportation routes in the two-echelon distribution network (due to the incurred fixed cost of transportation per vehicle) and increasing the allowable load of vehicles. In addition, this model attempts to minimize the CO_2_ emissions by increasing the number of vehicles and lowering their loads (the amount of CO_2_ emissions is directly related to the amount of loading). Also, the proposed model tries to increase job opportunities and economic growth by establishing more facilities. If the decision-makers concentrate on increasing social responsibility and reducing pollution, the first (cost) objective function increases significantly increases. Therefore, a trade-off solution should be provided for all of the three aspects of sustainability.

First, a small-sized problem was designed to validate the proposed multi-objective model. The model was solved using the exact AEC method together with two metaheuristic NSGA-II and MOSFS algorithms. Due to the NP-Hardness of the problem, the metaheuristic algorithms named NSGA-II and MOSFS were exploited through the MATLAB software to solve the large size problems and obtain the Pareto solutions. The results showed that the MOSFS algorithm obtained better Pareto frontier in terms of six indices. As some suggestions for future research, it is recommended to consider time dependencies and time windows for customers. Also, the reverse routes may be considered in the model. Moreover, customers can be categorized based on their consumption attitudes.

## Author contribution statement

Masoud Hajghani: Analyzed and interpreted the data; Contributed reagents, materials, analysis tools or data; Wrote the paper.

Mohammad Ali Forghani: Conceived and designed the experiments.

Ali Heidari: Performed the experiments; Analyzed and interpreted the data.

Mohammad Khalilzadeh: Analyzed and interpreted the data; Wrote the paper.

Omid Kebriyaii: Performed the experiments; Wrote the paper.

## Funding statement

This research did not receive any specific grant from funding agencies in the public, commercial, or not-for-profit sectors.

## Data availability statement

Data will be made available on request.

## Declaration of interest’s statement

The authors declare no competing interests.
